# Effect of the Surgical Safety Checklist on the incidence of adverse events: contributions from a national study

**DOI:** 10.1590/0100-6991e-20223286_en

**Published:** 2022-05-20

**Authors:** LUCIANE RIBEIRO DE FARIA, TIAGO RICARDO MOREIRA, FÁBIO DA COSTA CARBOGIM, RONALDO ROCHA BASTOS

**Affiliations:** 1 - Universidade Federal de Juiz de Fora, Enfermagem Aplicada - Juiz de Fora - MG - Brasil; 2 - Universidade Federal de Viçosa, Medicina e Enfermagem - Viçosa - MG - Brasil; 3 - Universidade Federal de Juiz de Fora, Estatística - Juiz de Fora - MG - Brasil

**Keywords:** Checklist, Patient Safety, Surgical Procedures Operative, Lista de Checagem, Segurança do Paciente, Procedimentos Cirúrgicos Operatórios

## Abstract

**Objective:**

the study evaluated the effect of using the safe surgery checklist (CL) on the incidence of adverse events (AE).

**Methods:**

cross-sectional and retrospective research with 851 patients undergoing surgical procedures in 2012 (n=428) and 2015 (n=423), representing the periods before and after CL implantation. The AE incidences for each year were estimated and compared. The association between the occurrence of AE and the presence of CL in the medical record was analyzed.

**Results:**

a reduction in the point estimate of AE was observed from 13.6% (before using the CL) to 11.8% (with the use of the CL). The difference between the proportions of AE in the periods before and after the use of CL was not significant (p=0.213). The occurrence of AE showed association with the following characteristics: anesthetic risk of the patient, length of stay, surgery time and classification of the procedure according to the potential for contamination. Considering the proportion of deaths, there was a significant reduction in deaths (p=0.007) in patients whose CL was used when compared to those without the use of the instrument. There was no significant association between the presence of CL and the occurrence of AE. It was concluded that the presence of CL in the medical record did not guarantee an expected reduction in the incidence of AE.

**Conclusion:**

however, it is believed that the use of the instrument integrated with other patient safety strategies can improve the safety/quality of surgical care in the long term.

## RESUMO

**Objetivo:**

o estudo objetivou avaliar o efeito da utilização do checklist (CL) de cirurgia segura na incidência de eventos adversos (EA).

**Método:**

pesquisa transversal e retrospectiva com 851 pacientes submetidos a procedimentos cirúrgicos nos anos de 2012 (n=428) e 2015 (n=423), representando os períodos antes e após a implantação do CL. As incidências de EA para cada ano foram estimadas e posteriormente comparadas. Também foi analisada a associação entre a ocorrência do EA e a presença do CL no prontuário.

**Resultados:**

observou-se uma redução na estimativa pontual de EA de 13,6% (antes do uso do CL) para 11,8% (com a utilização do CL). No entanto, a diferença entre as proporções de EA nos períodos antes e após a utilização do CL não foi significativa (p=0,213). A ocorrência do EA mostrou associação significativa às seguintes características: risco anestésico do paciente, tempo de internação, tempo de cirurgia e classificação do procedimento segundo o potencial de contaminação. Considerando a proporção de óbitos ocorridos nas amostras, observou-se uma redução significativa de mortes (p=0,007) em pacientes cujo CL foi utilizado quando comparados aqueles sem o uso do instrumento. Não foi verificada associação significativa entre a presença do CL no prontuário e a ocorrência do EA de forma geral.

**Conclusão:**

a presença do CL no prontuário não garantiu uma redução esperada na incidência de EA. No entanto, acredita-se que o uso do instrumento integrado às demais estratégias de segurança do paciente possa melhorar a segurança/qualidade da assistência cirúrgica em longo prazo.

## INTRODUCTION

The importance of surgical treatment is recognized worldwide due to the great benefits provided to patients, such as the cure of many diseases and the reduction of morbidity and mortality. Despite this, safety failures in surgical procedures can cause significant harm and a great impact on the quality of life of patients and their families^1^. Research information on complications associated with surgical care show a high frequency of this type of harm^2^ ^,^ ^3^. The consequences of these events can translate into temporary impairments, permanent physical disabilities, and even deaths. In addition, it is necessary to consider the increase in treatment costs due to prolonged hospitalization and the need for new therapies/interventions^2^ ^-^ ^5^.

The magnitude of harm associated with health care (adverse events - AE) attracted the attention of the World Health Organization (WHO), which in 2004 launched the World Alliance for Patient Safety. One of the challenges proposed by the Alliance had, as its motto, “Safe Surgery Saves Lives”^6^. To meet this challenge, the WHO recommended the adoption of a Surgical Safety Checklist (checklist or CL)^6^. After carrying out a pilot study to validate the standard instrument proposed by the WHO^7^, the adoption of the CL was strongly encouraged, as well as its adaptation according to the reality of health organizations.

With the increasing adherence to the CL use, several international studies have been dedicated to evaluating the effect of using this tool in surgical care. Some studies have found benefits attributed to its adoption, identifying a significant reduction in the incidence of AE^8^ ^-^ ^11^. Despite this, a systematic review found inconsistent results, which was related to the quality and methodological differences used by the studies. Though revealing the existence of uncertainties about the CL effect, the study concluded that the use of the instrument may be associated with the reduction of AE^12^.

As this is a recently implemented technology, little is known about the effect of using the CL^13^, mainly in developing countries, especially in Brazil. So far, national studies show non-compliance in its filling and low completeness of check items^14^ ^-^ ^17^.

In this context, the production of information that can elucidate the effect of CL in surgical care, discuss its clinical effectiveness, and support its use is of great importance. Thus, the present study aimed to evaluate the effects of using the Surgical Safety Checklist on the incidence of AE in a referral hospital located in the interior of the State of Minas Gerais, Brazil.

## METHOD

We conducted a cross-sectional, retrospective study by reviewing the medical records of patients undergoing surgical procedures of all specialties. The scenario was a high complexity reference general hospital, located in a municipality in the interior of Minas Gerais, which performs an average of 1,500 surgical procedures per month.

The CL was implemented at the institution in the first half of 2013, being an adaptation of the WHO standard instrument, comprising 19 check items. Initially, all surgical patients from the years 2012 (before using the CL) and 2015 (after using the CL) were considered eligible. Exclusion criteria were age below 18 years, hospitalization period of less than 24 hours, patients who underwent non-surgical invasive procedures, interventional cardiology procedures, and vaginal delivery.

To calculate the sample, we considered a total of 6,201 surgical patients for the year 2012 and 6,158 for the year 2015, a test power of 80%, a standardized difference between the proportions of patients with AE in the years 2012 and 2015 equal to 0.20^18^, the same size for each sample, with unknown population variances, but equal and independent samples. Thus, the sample size calculation for α=0.05 indicated the need to evaluate the medical records of at least 786 surgical patients. The study considered 428 patients for the year 2012 and 423 for the year 2015, totaling 851 patients.

The sample of medical records was extracted from a database made available by the institution. Initially, the database was organized considering the month in which the surgical procedure was performed. Next, the medical records were selected by simple random sampling, being proportional to the number of surgeries performed each month to allow monitoring of the incidence of AE over time.

AE tracking and identification were guided by an adaptation of the Global Trigger Tool (GTT) method proposed by the Institute for Healthcare Improvement (IHI), which presents objective criteria/ triggers for tracking records with suspected AEs^19^. As a definition for AE, the one described by the GTT was adopted, as an unintentional physical damage resulting directly or indirectly from health care, which requires additional monitoring, treatment, or hospitalization, or even which resulted in death^19^. The review of medical records was carried out from January to December 2019, by a nurse and three undergraduate students. The procedure of double review of medical records was adopted independently. The team of reviewers was joined by two physicians with expertise in the use of the GTT method, who acted as authenticators of the occurrence of the AEs and the classification of their severity.

Regarding the classification of AE severity, the IHI recommends that it be performed as follows: E) temporary harm to the patient that required intervention; F) Temporary harm to the patient that required additional intervention or prolonged hospitalization; G) permanent harm to the patient; H) harm that required immediate intervention to save the patient’s life; and I) death^19^.

We computed and compared the AE incidences corresponding to each year surveyed. The primary outcome chosen was the occurrence of AE, whose choice lies on the understanding that the use of the CL can improve surgical safety, both directly (considering the checking of specific items present in the instrument, such as the identification of the patient and the correct surgical site) and indirectly (increased patient safety culture in the health organization, contributing to the reduction of any type of AE). In addition, the incidence of AE was the primary outcome chosen in most studies on the subject^20^ ^-^ ^22^.

The independent variables investigated were: patient characteristics, such as sex, age, age-corrected Charlson Comorbidity Index (CCI) score^23^, and anesthetic risk according to the American Society of Anesthesiology (ASA) classification; characteristics of the hospitalization, such as type of care, character of hospitalization, length of stay, and reason for leaving; characteristics of the surgical procedure, such as specialty, surgery shift, surgery time, and classification of the surgery in terms of urgency and potential for contamination; and CL characteristic, such as presence of the instrument in the medical record.

The initial analysis included a description of the study variables through descriptive statistics and exploratory data analysis. Bivariate analysis investigated the association of the outcome with independent variables, using the Pearson’s chi-square test at a significance level of 5%. We assessed the difference between the proportions of AE in the medical records samples referring to the periods before and after the use of CL using the Student’s T test for independent samples. We studied the magnitudes of the association between the outcome and the independent variables through the estimation of parameters of multivariate logistic regression models, in the statistical package Statistical Package for the Social Sciences (SPSS, version 20.0 for Windows).

The research project was approved by the Ethics in Research Committee of the Federal University of the Municipality, under protocol number 2,046,497.

## RESULTS

The study sample included 851 medical records of surgical patients. Considering only the sample of records belonging to the period in which the CL was used (n=423), the instrument was present in 95% of the analyzed records. The instrument was complete (all items checked) in 67.4% of these records.

The characteristics of the patients (Table 1) in the two periods studied was similar in relation to age group, score according to the CCI, and anesthetic risk according to the ASA classification. Most were in the range aged up to 59 years and had an CCI score of up to 1, indicating that comorbidities, when present, were mild and did not cause limitations. Regarding anesthetic risk, most were classified as ASA-1 and ASA-2, reflecting the same clinical conditions evidenced by the CCI, that is, healthy patients or patients with mild and controlled diseases.

**Table 1:** Characteristics of the patients, hospitalization, and surgical procedure, before and after the use of the Surgical Safety Checklist.

Variables	Before CL (n=428)		After CL (n=423)		p-value
	n	%	n	%	
Patient characteristics					
Age Group					0.814
up to 59 years	278	65.0	278	65.7	
60 years +	150	35.0	145	34.3	
Sex					0.003
Male	143	33.4	183	43.3	
Female	285	66.6	240	56.7	
CCI					0.816
0	188	43.9	177	41.9	
1	53	12.4	48	11.4	
2-3	98	22.9	101	23.8	
4+	89	20.8	97	22.9	
Variables	Before CL (n=428)		After CL (n=423)		p-value
	n	%	n	%	
Anesthetic risk					0.073
ASA-1	151	35.3	181	42.8	
ASA-2	196	45.8	166	39.3	
ASA-3	68	15.9	69	16.3	
ASA-4	13	3.0	7	1.6	
Characteristics of hospitalization					
Type of service					0.067
SUS	213	49.8	237	56.0	
agreement or private	215	50.2	186	44.0	
Type hospitalization					0.021
Elective	219	51.2	183	43.3	
Emergency	209	48.8	240	56.7	
Length of stay in days					0.986
<2	179	41.8	175	41.4	
3	49	11.5	52	12.2	
4-10	96	22.5	94	22.2	
11+	104	24.2	102	24.2	
Outcome					0.140
Discharge	400	93.5	405	95.7	
Death	28	6.5	18	4.3	
Features of surgery					
Specialty					0.288
General surgery	108	25.2	85	20.0	
Gynecology and Obstetrics	98	22.9	103	24.4	
Orthopedics and traumatology	81	18.9	100	23.7	
Cardiothoracic and vascular surgery	58	13.6	57	13.5	
Others	83	19.4	78	18.4	
Surgery shift					0.044
Morning	193	45.1	160	37.8	
Afternoon	167	39.0	173	40.9	
Night	68	15.9	90	21.3	
Type of anesthesia					0.033
Sedation/local	33	7.7	18	4.3	
Variables	Before CL (n=428)		After CL (n=423)		p-value
	n	%	n	%	
Regional	212	49.5	239	56.5	
General	183	42.8	166	39.2	
Surgery time in minutes					0.000*
≤30	56	13.1	26	6.1	
31-60	131	30.6	153	36.2	
61-120	124	29.0	146	34.5	
121-140	79	18.5	81	19.1	
241+	38	8.9	17	4.0	
Contamination Potential					0.925
Clean	201	47.0	194	45.9	
Potentially Contaminated	175	40.9	172	40.7	
Contaminated	30	7.0	31	7.3	
Infected	22	5.1	26	6.1	
Urgency					0.132
Elective	326	76.2	303	71.6	
Emergency	102	23.8	120	28.4	

Regarding hospitalization (Table 1), the length of stay was similar in both groups, most patients having a length of stay of less than two days (41.8% and 41.4%, respectively). As for the surgical procedure, both groups had a greater proportion of patients undergoing surgery lasting up to 60 minutes (p<0.001) and using regional anesthesia (p=0.033).

**Figure 1:**
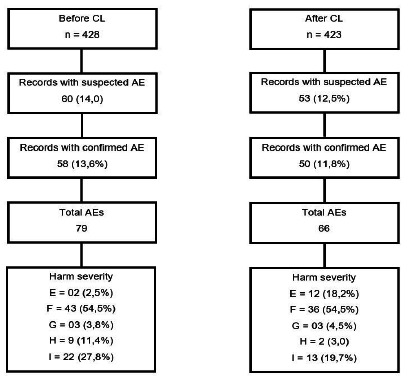
Flowchart of tracking of adverse events in the surgical patients’ sample in the periods before and after the use of the CL.

After completing the AE tracking and identification step, as described in Figure 1, we compared the groups referring to the periods before and after the CL use. We observed a reduction in the point estimate of AE incidence from 13.6% to 11.8%, without, however, significant differences between the proportions (p=0.438). Regarding the severity of harm, the percentage of those classified as light and temporary (category E) increased between the analyzed periods (2.5% and 18.2%, respectively), while the percentages included in categories F (54.5% in both samples) and G (3.8% and 4.5% respectively) remained stable. As for death (category I), there was a reduction in the percentage from 27.8% before the use of the CL to 19.7% in the period when it was used.

The description of the AEs identified in the sample (Table 2) revealed that harm related to the surgical site was the most frequent in both periods, with emphasis on the occurrence of surgical site infection (SSI) and bleeding with hemodynamic repercussions. When considering AEs not related to the surgical wound, infections with a pulmonary focus were the most frequent in the periods studied. It is noteworthy that the percentage of AE related to the surgical site increased between the periods before and after the CL implementation (43.1% to 60.6%, respectively), while infections not related to the surgical wound and the cardiovascular complications decreased (26.6% to 24.2% and 12.7% to 7.5%, respectively).

**Table 2:** Proportion of adverse events in the sample of surgical patients before and after the CL adoption.

	Before CL		After CL		Total	
	n	%	n	%	n	%
Related to the surgery						
Surgical site infection	10	12.7	11	16.7	21	14.5
Bleeding with hemodynamic repercussions	10	12.7	14	21.2	24	16.6
Iatrogenic injury to other organs or tissues	3	3.8	5	7.6	8	5.5
Fistula	3	3.8	5	7.6	8	5.5
Peripheral nerve injury	1	1.3	2	3.0	3	2.0
Incisional hernia	2	2.5	-	-	2	1.8
Suture dehiscence	2	2.5	1	1.5	3	2.0
Others	3	3.8	2	3.0	5	3.4
Subtotal	34	43,1	40	60,6	74	51,0
Infections unrelated to the surgical wound						
Pulmonary focus	12	15.4	8	12.2	20	13.9
Urinary focus	3	3.7	4	6.0	7	4.9
Abdominal focus	2	2.5	1	1.5	3	2.0
Unknown focus	2	2.5	2	3.0	4	2.7
Others	2	2.5	1	1.5	3	2.0
Subtotal	21	26.6	16	24.2	37	25.5
Cardiovascular complications						
Acute myocardial infarction	1	1.4	2	3.0	3	2.0
Pulmonary thromboembolism	3	3.8	-	-	3	2.0
Deep vein thrombosis	2	2.5	-	-	2	1.8
Acute lung edema	2	2.5	1	1.5	3	2.0
	Before CL		After CL		Total	
	n	%	n	%	n	%
Cardiogenic shock	2	2.5	2	3.0	4	2.8
Subtotal	10	12.7	5	7.5	15	10.4
Other complications	14	17.6	5	7.7	19	13.1
Total	79	100	66	100	145	100

In an additional division, where the patients in the global sample were separated by the presence or absence of CL in the medical record, we found that the proportion of deaths among patients whose CL was used was lower than among those who were not exposed to CL. (p=0.007), as shown in Table 3.

**Table 3:** Difference between the proportions of adverse events and deaths with and without the Checklist in the medical records of surgical patients.

	T	p-value	Proportions’ Difference	95% CI
AE	1.25	0.213	0.028	-0.16 - 0.073
Death	2.72	0.007	0.041	0.011 - 0.071

The multivariate analysis showed that the presence of CL in the surgical patient’s medical record was not significantly associated with the occurrence of AE (p=0.622), suggesting that AE is more associated with the procedure characteristics, since these variables also remained significant when controlled by the presence of CL (Table 4).

**Table 4:** Multivariable analysis of the outcome Occurrence of an adverse event in the surgical patients, for the years 2012 and 2015.

Variables	β	p-value	OR	95% CI
Anesthetic risk				
ASA-1	-	-	1.00	-
ASA-2	1.39	0.001	4.00	1.74 - 9.25
ASA-3	1.04	0.030	2.83	1.11 - 7.21
ASA-4	1.26	0.082	3.50	0.86 - 14.32
Length of stay				
≤2 days	-	-	1.00	-
3 days	0.17	0.817	1.27	0.32 - 5.07
4-11 days	1.05	0.020	3.01	1.19 - 7.61
11 days +	1.86	0.000 *	7.43	2.90 - 19.05
Classification of the surgery according to the potential for contamination				
Clean	-	-	1.00	
Potentially contaminated	- 0.147	0.663	0.87	0.45 - 1.67
Contaminated	1.20	0.007	3.36	1.40 - 8.08
Infected	0.80	0.089	2.25	0.89 - 5.70
Surgery time				
≤30 min	-	-	1.00	-
31-60 min	0.26	0.696	1.30	0.36 - 4.70
61-120 min	0.79	0.228	2.20	0.62 - 7.83
Variables	β	p-value	OR	95% CI
121-240 min	1.03	0.125	2.80	0.76 - 10.39
240 min +	2.08	0.004	7.97	1.97 - 32.34
Checklist presence				
Absent	-	-	1.00	-
Present	0.13	0.622	1.14	0.68 - 1.92

## DISCUSSION

The use of the Surgical Safety Checklist has been a strongly recommended strategy by the WHO because it is considered an effective intervention, relatively easy to apply, with low cost, and with the potential to reduce complications and deaths associated with surgical care^6^. In addition, studies on the use of the instrument have shown that adherence to safety checks contributes to the development of a safety culture in health organizations, valuing interdisciplinary work and improving communication between team professionals^9^ ^,^ ^12^.

Considering the increasing frequency and severity of the damage produced by AEs related to surgical care, added to the promising results revealed by the pioneering study on the benefits brought by applying the CL^7^, there is a continuous effort around the world to adopt the instrument in surgical care and progressively improve adherence. In Brazil, since the publication of RDC Nº36/201324, health organizations have also moved towards inserting surgical safety checking as a strategy to improve safety levels and raise health care quality standards.

In view of the strong WHO recommendations for the use of the CL for safe surgery, there was a need to seek results on the effectiveness of the use of this tool. From this perspective, many studies have found specific benefits attributed to the CL use in developed countries, demonstrating a significant reduction in AE incidence, as in Norway (19.9% to 11.5%; p=0.001)^8^ and England (16.9% to 11.2%; p=0.01)^9^. However, other studies showed different results, stating that the use of CL did not contribute to a significant reduction in complications associated with surgical care, as in Canada (3.86% to 3.82%; p=0.53)^25^ and Spain (18.1% to 16.2%; p=0.35)^10^. Also in this context, retrospective studies conducted in American hospitals found controversial results. While one of them showed a significant reduction in AE incidence, from 23.6% to 8.2% (p=0.000)^26^, the other showed that the introduction of CL did not contribute to the reduction of complications in surgical care (p=0.799)^27^. Even considering the different methodological approaches, the benefits brought by using the CL are still not well elucidated by the available studies, indicating the need to continuously monitor the impact of the instrument use in surgical care^13^.

In our study, the analysis by the logistic regression model revealed that the presence of CL in the medical record did not present a significant association with the occurrence of AE (p=0.622), despite the drops in point estimates found in both the incidence and severity of the events, considering the periods before and after CL use. Systematic reviews that sought to assess the effect of the CL on the occurrence of surgical complications showed that in developing countries the positive results are lower than those observed in developed countries, demonstrating that in these scenarios, the use of the instrument has not yet provided the expected effect on patient safety^12^ ^,^ ^28^ ^-^ ^30^.

It is important to emphasize that in this study, the CL was present in 95% of the analyzed medical records. However, it was complete in only 67.4% of the them^17^. Thus, the impact of using the CL in surgical care may be compromised by the incompleteness of the instrument, signaling the need for improvement in the development of the patient safety culture in the institution. The introduction of a document in the care of the surgical patient by itself is not enough to guarantee a reduction in surgery-related complications. It is essential that the health organization prioritizes management based on values, skills, and behaviors that encourage the commitment of all employees to safety in health care. In addition, the immediate effect of using the CL may not be the same for all situations, as suggested by the WHO, considering the different political, socioeconomic, and cultural contexts^12^ ^,^ ^22^ ^,^ ^30^.

Regarding the significant reduction (p=0.007) in the proportion of deaths that occurred between the two periods studied, the result is considered as indicative of a possible improvement in the quality of surgical care, since this difference reflects a reduction in the proportion of AEs with greater severity. Significant reductions in the occurrence of deaths before and after CL implantation were also found in developed countries, such as Australia (1.2% to 0.92%; p=0.038)^11^, and in developing ones, such as India (10 % to 5.7%; p=0.004)^29^. In the present study, it is important to highlight that most patients in the groups before and after CL implantation were in the age range of up to 59 years and had mild comorbidities, when present. Therefore, we believe that the profile of patients regarding the risk of death did not influence the result found.

Some important issues related to the implementation process and use of the CL in the study scenario may be related to the results found. The implementation of the safe surgery protocol was an initiative of the Patient Safety Center, with the participation of nurses from the Operating Room, who were responsible for adapting the CL. Regarding training on the importance and proper use of the instrument, only the nursing team was involved, with no training of the medical team (surgeons, anesthesiologists, and residents). Another issue that needs to be pointed out refers to the adaptation of the instrument, which excluded some of the standard CL check items proposed by the WHO. Adaptation is recommended to improve adherence to the instrument due to cultural differences. However, the exclusion of previously validated check items is not encouraged6. All these issues may reflect on the way the instrument has been used in practice. Despite being present in most medical records, completeness is below ideal, demonstrating weaknesses in its use^17^.

The study also has limitations inherent to its design. The identification of the AE from the retrospective review of medical records depends directly on the quality of the records, which can contribute to underestimation of the cases. In addition, the use of clinical judgment to define AE occurrence and classify its severity may be influenced by the subjectivity of the physicians who participated in this phase of the study. However, this has been the most used method in most studies on the same topic, and there is no other method considered as the best available evidence for the identification of AEs. Additionally, the analysis of the CL effect two years after its implementation may not have contemplated the time needed to assess the instrument’s consolidation in surgical practice 

## CONCLUSION

The present study offers an important contribution, as it is a national assessment of the effect of using the CL for safe surgery, since knowledge on the subject is quite incipient. The evaluation carried out here has important value for showing evidence on the CL use in the context of a developing country, where human, material, and structural resources, together with technological advances, are below those found in developed countries.

The results found here showed that the presence of CL in the medical records of surgical patients was not associated with a reduction in AE occurrence in general. Thus, the expected benefits from the introduction of the instrument could not yet be confirmed by the evidence presented here. However, the significant reduction in the proportion of deaths observed after the CL implementation suggests that the use of the instrument in surgical practice may be contributing to the reduction of more severe AEs. The positive changes in surgical care should not be attributed solely to the CL adoption, though the implementation of the instrument in the routine of a health organization already represents an initiative for the development of a patient safety culture.

## References

[1] Weiser TG, Regenbogen SE, Thompson KD, Haynes AB, Lipsitz SR, Berry WR (2008). An estimation of the global volume of surgery a modelling strategy based on available data. Lancet.

[2] Batista J, Cruz EDA, Alprende FT, Rocha DJM, Brandão MB, Maziero ECS (2019). Prevalência e evitabilidade de eventos adversos cirúrgicos em hospital de ensino do Brasil Rev. Latino-Am. Enfermagem.

[3] Anderson O, Davis R, Hanna GB, Vicent CA (2013). Surgical adverse events a systematic review. Am J Surg.

[4] Moura MLO, Mendes W (2012). Avaliação dos eventos adversos cirúrgicos em hospitais do Rio de Janeiro. Rev Bras Epidemiol.

[5] Nilsson L, Risberg BM, Montgomery A, Sjodahl R, Schildmeijer K, Rutberg H (2016). Preventable adverse events in surgical care in Sweden - A Nationwide review of patient notes. Medicine (Baltimore).

[6] Organização Mundial de Saúde (2009). Segundo desafio global para a segurança do paciente: cirurgia segura salvam vidas (orientações para cirurgia segura da OMS).

[7] Haynes AB, Weiser TG, Berry WR, Lipsitz SR, Breizat AHS, Dellinger EP (2009). A surgical safety checklist to reduce morbidity and mortality in a global population. N Engl J Med.

[8] Haugen AS, Softeland E, Almeland SK, Sevdalis N, Vonen BV, Eide GE (2015). Effect of the World Health Organization checklist on patient outcomes a stepped wedge cluster randomized controlled trial. Ann Surg.

[9] Mayer EK, Sevdalis N, Rout S, Caris J, Russ S, Mansell J (2015). Surgical Checklist Implementation Project the Impacto of Variable WHO Checklist Compliance on Risk-adjusted Clinical Outcomes After National Implementation: A Longitudinal Study. Ann Surg.

[10] Rodrigo-Rincon I, Martin-Vizcaino MP, Tirapu-Leon B, Zabalza-Lopez P, Zaballos-Barcala N, Villalgordo-Ortin P (2015). The effects of surgical checklists on morbidity and mortality a pré- and post-intervention study. Acta Anaesthesiol Scand.

[11] Jager E, Gunnarsson R, Yik-Hong H (2018). Implementation of the World Health Organization Surgical Safety Checklist correlates with reduced surgical mortality and length of hospital admission in a high-income country. World J Surg.

[12] Jager E, McKenna C, Bartlett L, Gunnarsson R, Yik-Hong H (2016). Postoperative adverse events inconsistently improved by the world Health Organization Surgical Safety Checklist a systematic literature review of 25 studies. World J Surg.

[13] Abbott TEF, Ahmad T, Phull MK, Fowler AJ, Hewson R, Biccard BM (2018). The surgical safety checklist and patient outcomes after surgery a prospective observational cohort study, sistematic review and meta-analysis. Br J Anaesth.

[14] Ribeiro HCTC, Quites HFO, Bredes AC, Sousa KAS, Alves M (2017). Adesão ao preenchimento do checklist de segurança cirúrgica Cad. Saúde Pública.

[15] Campos AL, Santos RP (2017). Use of safe surgery checklist in Brazilian health services integrative review. Int J Dev Res.

[16] Leite GR, Martins MA, Maia LG, Garcia-Zapata MTA (2020). Checklist de cirurgia segura avaliação em uma região neotropical. Rev. Col. Bras. Cir.

[17] Ribeiro L, Fernandes GC, Souza EG, Souto LC, Santos ASP, Bastos RR (2019). Checklist de cirurgia segura adesão ao preenchimento, inconsistências e desafios. Rev. Col. Bras. Cir.

[18] Machin D, Campbell M, Fayers P, Pinol A (1997). A sample size tables for clinical studies.

[19] Griffin F, Resar R (2009). IHI Global Trigger Tool for Measuring Adverse Events.

[20] Haynes AB, Weiser TG, Berry WR, Lipsitz SR, Breizat AS, Dellinger EP (2011). Changes in safety attitude and relationship to decreased postoperative morbidity and mortality following implementation of a checklist-based surgical safety intervention. BMJ Qual Saf.

[21] Rodella S, Mall S, Marino M, Turci G, Gambale G, Montella MT (2018). Effects on clinical outcomes of a 5-year surgical safety checklist implementation experience a large-scale population-based difference-in-differences study. Health Serv Insights.

[22] Collins SJ, Newhouse R, Porter J, Talsma A (2014). Effectiveness of the Surgical Safety Checklist in correcting errors a literatura review applying reason's swiss cheese model. AORN Journal.

[23] Martins M (2010). Uso de medidas de comorbidades para predição de risco de óbito em pacientes hospitalizados. Rev Saúde Pública.

[24] Ministério da Saúde. Agência Nacional de Vigilância Sanitária (2013). Resolução RDC nº36, de 25 de julho de 2013. Institui ações para segurança do paciente em serviços de saúde e dá outras providências.

[25] Urbach DR, Govindarajan A, Saskin R, Wilton AS, Baxter NN (2014). Introduction of surgical safety checklists in Ontario, Canada. N Engl J Med.

[26] Biskup N, Workman AD, Kutzner E, Adetayo OA, Gupta SC (2016). Perioperative safety in plastic surgery is the World Health Organization Checklist useful in a broad practice?. Ann Plast Surg.

[27] Bliss LA, Ross-Richardson CB, Sanzari LJ, Shapiro DS, Lukianoff AE, Bernstein BA (2012). Thirty-day outcomes support implementations of a surgical safety checklist. J AM Coll Surg.

[28] Vivekanantham S, Ravindran RP, Shanmugarajah K, Maruthappu M, Shalhoub J (2014). Surgical safety checklists in developing countries. Int J Surg.

[29] Prakash P, Baduni N, Sanwall MK, Sinha SR, Shekhar C (2014). Effect of World Health Organization surgical safety Checklist on patient outcomes in a Tertiary Care Hospital of Delhi. Int Med J.

[30] Gama CS, Backman C, Oliveira AC (2019). Effect of surgical checklist on colorectal surgical site infection rates in 2 countries Brazil and Canada. Am J Infect Control.

